# The Treatment of Pneumococcic Infection

**Published:** 1904-04-16

**Authors:** 


### Hospital Clinics and Medical Progress.

### THE TREATMENT OF PNEUMOCOCCIC INFECTION.

In a lecture delivered on this subject at the hospital of the University of Pennsylvania Dr. John H. Musser[1] claims that the designation "pneumococcic infection" is preferable to "croupous pneumonia" or "lobar pneumonia" for the reason that the inflammatory lesions in the lung may be the smallest part of the disease process going on in the body, and because the extent of the lung involvement has not much to do with the severity of the patient's condition. He directs attention to the importance of bearing in mind that the local lesions in the lung are accountable for some, but not for all, of the patient's symptoms; that the patient ill with croupous pneumonia is suffering from a general infection with the pneumococcus; that organs other than the lungs may reveal morbid lesions at least as well marked as the local pulmonary lesions; that in some cases these other lesions are susceptible of recognition; and that the severity of the clinical manifestations in a given case depends upon the virulence of the infecting pneumococcus and the resistance offered by the patient. Thus the extent of the local lesions, be they in the lung or elsewhere, bears no necessary relation to the severity of the general symptoms, which are merely the expression of a general toxæmia. The greater the virulence of the infecting micro-organism, and the less the tissues of the patient are able to neutralise the toxins, the greater is the resulting toxæmia. Experience has taught that the dangers of pneumonia are due almost entirely to the toxæmia, and that in 90 per cent. of fatal cases death results from toxic poisoning and not from the amount of lung involvement or mechanical embarrassment of the circulation. In pneumonia, as in all diseased conditions, it is necessary to remember that there is a patient to be treated rather than an abstract illness, and modifications will have to be made according to the peculiarities of the patient and exigencies of the case. The exact treatment in individual cases must be based on, (1) the diagnosis, (2) a mental concept of the morbid process, (3) the symptoms, (4) the results of an examination of the different organs of the body, and (5) suggestive facts elicited from the family history and previous history of the patient. Proceeding upon this plan, there is only one course suggested by the mere diagnosis alone, and that is the use of anti-pneumococcic serum. This Dr. Musser has sufficient experience of to be able to say that it does no harm, though he does not think that it hastens the crisis or influences the extent of the local inflammatory lesions. As it may do some good its use is quite justified. In the case of syphilis, diphtheria, and malaria the use of the specific remedy—mercury and iodides, antitoxin, or quinine as the case may be—is at once indicated by the diagnosis; but unfortunately a specific remedy for pneumonia has yet to be found.

Treatment based upon a knowledge of the morbid process, taking that to be a general infection with the pneumococcus and a local inflammatory lesion in the lung as already indicated, will be directed (1) toward arresting, limiting, or modifying the extent of the local inflammatory lesions in the lung; (2) to assisting nature to eliminate the toxins; and (3) towards counteracting the effects of the toxins. When a case is seen early it is well to aim at lowering the blood pressure in order to diminish the pulmonary congestion. A good way to do this is to give 10 grains of calomel. This acts both as a laxative and as a local antiseptic. In order to utilise the latter property the calomel should be placed dry upon the tongue, and the patient asked not to swallow it at once but to let it mix with the salivary secretion. In this way the mouth, fauces, and upper respiratory passages may be to some extent freed from the presence of the virulent pneumococci which are usually to be found in these situations in cases of pneumonia. The pulmonary congestion may further be controlled by local applications such as dry cupping. Dr. Musser uses 20 or 30 cups at a time, and finds that they relieve pain very considerably, and also relieve the breathing so that respirations will slow down from say 35 per minute to 30. He finds the application of cold, as by an ice-bag, or by the application of a cotton jacket wrung out of cold water, to be of additional service. He is confident that he has witnessed "the greatest amount of good follow this method of stimulating the cardiac and respiratory mechanisms by what may be looked upon as the heroic application of cold."

There is now considerable evidence, both clinical and experimental, to show that the degree of toxæmia in an infective disease depends upon the activity of the kidneys. This is encouraged by maintaining the strength of the heart and giving large quantities of water, with or without saline diuretics. For the heart $\frac{1}{30}$ to $\frac{1}{40}$ grain of strychnine may be given every four hours, while the kidneys are stimulated by large draughts of water—two or three quarts being given in the 24 hours. If these measures do not suffice, and renal secretion lessens while the pulse becomes weak, the dose of strychnine should be increased to $\frac{1}{20}$ grain, and a saline injection given by the rectum, to be followed by subcutaneous slow injection of large quantities of normal saline solution. The latter is one of the most serviceable means that exist of treating pneumonia. In almost every case hypodermoclysis is followed by marked improvement in the condition of the patient. Dr. Musser rarely uses alcohol, and never if he can avoid it; he reserves its use for alcoholic subjects and for other patients when the question arises of tiding them over a critical period of, say, 12 or 24 hours.

With regard to the treatment of symptoms: for pain in the early stages opium may be given, but not after the first 24 or 48 hours. Signs suggestive of meningitis may occur. These may or may not be due to infection of the meninges. If there is serious doubt lumbar puncture should be performed, both to assist the diagnosis and to relieve the patient. If the circulation is failing good results attend the use of nitroglycerin, if there is high blood-pressure, or digitalis if the blood-pressure is low. If there are signs of engorgement of the right heart (precordial distress, cyanosis, etc.) phlebotomy may prove exceedingly useful. In cases where there is much associated bronchitis, especially in old people, atropin in small doses may be of much benefit in diminishing the secretion. Dr. Musser lays great stress on the importance of watching the intestinal condition. He is satisfied that in many cases death results from extreme tympanites interfering with cardiac and respiratory action. Upon the first evidence of abdominal distension, therefore, moderate purgation should be produced and the lower bowel washed out.

[1] International Clinics, Vol. IV., Series 13.

